# INTEREST GROUP SESSION—PATIENT/PERSON ENGAGEMENT IN RESEARCH INTEREST GROUP (PPER-IG): STRENGTH IN AGE: ENGAGING OLDER ADULTS AS HEALTH RESEARCHERS THROUGH COMMUNITY-BASED PARTNERSHIPS

**DOI:** 10.1093/geroni/igz038.1500

**Published:** 2019-11-08

**Authors:** Silvia Sörensen, Rebecca S Allen, Reza Yousefi Nooraie

**Affiliations:** 1 University of Rochester Warner School for Education and Human Development, Rochester NY, United States; 2 The University of Alabama, Tuscaloosa, Alabama, United States; 3 URMC, University of Rochester School of Medicine and Dentistry, New York, United States

## Abstract

The lack of clear translation of health research to improving older under-served patients’ lives presents a serious problem. Studies of aging rarely include the older adults themselves in the process of conceptualizing questions, implementing the research, and applying and evaluating the results. Lack of input particularly from marginalized and minority older adults may compromise the relevance and accuracy of health research findings. In this symposium, we present the design and evaluation of two projects funded by the Patient-Centered Outcomes Research Institute (PCORI), in which older adults are trained to understand research language, culture, and methods, and are subsequently incorporated into research projects in a variety of roles. Silvia Sörensen will describe the “Engaging Older Adult Learners as Health Researchers” (ENGOAL) in Rochester, NY. This program provides six months of weekly classes and 4-6 months of research apprenticeships for older adults. Dorine Otieno and Kate Kondolf will describe evaluation results from both quantitative and qualitative analyses. Rebecca Allen will describe the design and implementation of “Sharing Opinions and Advice about Research (SOAR) in the Deep South,” a partnership of The University of Alabama with community stakeholders from Sumter and Holt County to recruit and train community members to assist in the formulation of research questions based on the needs of their communities. Allen and Dragan will present the evaluation results from this project with regard to implementation and graduate education. Reza Yousefi-Nooraie will synthesize the insights from these projects and add the perspective of a social network analyst.

